# Preparedness of Residents to Manage Pediatric Nonalcoholic Fatty Liver Disease: A National Survey

**DOI:** 10.1097/PG9.0000000000000219

**Published:** 2022-06-21

**Authors:** Namrata Patel, Valentina Discepolo, Nour Asfour, Ruba K. Azzam

**Affiliations:** * Department of Pediatric Gastroenterology, Hepatology and Nutrition, University of California, San Francisco, San Francisco, CA; † Department of Translational Medical Sciences, Section of Pediatrics, University of Naples Federico II, Naples, Italy; ‡ University of Chicago Pritzker School of Medicine, Chicago, IL; § Department of Pediatrics, Pediatric Gastroenterology, Hepatology & Nutrition, University of Chicago, Chicago, IL.

**Keywords:** NAFLD, pediatric residents, training, obesity

## Abstract

Nonalcoholic fatty liver disease (NAFLD) is reported to be the most common chronic pediatric liver disease. Little information is available on the adherence of residents in-training to the published guidelines for the evaluation and management of pediatric NAFLD. The goals of this study are as follows: (i) to assess the consistency of screening and evaluation for NAFLD in obese and overweight children at continuity clinics by upper level residents, and (ii) to determine the residents’ extent of training, knowledge, comfort, and competence levels in NAFLD care.

**Methods:**

An electronic survey developed using REDCap was emailed to accredited Pediatric Residency Programs in the United States. Program directors and coordinators were requested to forward the survey to their upper level pediatric and medicine/pediatrics residents. Statistical analysis of responses (n = 399) was performed.

**Results:**

More than 88% of residents reported to be exposed to obese and overweight children, representing at least 25% of the patients encountered in clinics. Regardless of their training level, they inconsistently screened for (>60%), initiated evaluation of, or provided counseling on NAFLD in these patients, not following the North American Society for Pediatric Gastroenterology, Hepatology and Nutrition guidelines. Over 80% of residents perceived to have received inadequate training resulting in insufficient knowledge on NAFLD, which they identified as their biggest barrier (25.7%). There was minimal statistically significant difference in the survey findings between training levels (PGY-2 vs PGY-3/4).

**Conclusions:**

Educational interventions should be implemented by pediatric residency programs to enhance educational core curricula for the early detection and initiation of management of NAFLD, an emerging public health problem.

**What Is Known**

- Nonalcoholic fatty liver disease (NAFLD) is the most common chronic pediatric liver disease, yet it is often undiagnosed.
- Screening at-risk children is crucial since early lifestyle modification can effectively reverse its progression.
- Both pediatric and adult guidelines delineate indications for pediatric NAFLD screening, evaluation, and management.

**What This Study Adds**

- This study provides insight into in-training residents’ practices in screening and early management of children with suspected NAFLD.
- It uncovers their perceived lack of knowledge and inadequate training.
- Results call for comprehensive NAFLD educational interventions during pediatric residency.

## INTRODUCTION

Nonalcoholic fatty liver disease (NAFLD) is the most common chronic liver disease in adults as well as in children in the United States of America (USA) (1). The pooled mean prevalence of pediatric NAFLD was 7.6% (95% confidence intervals [CI], 5.5-10.3) in general population studies and 34.2% (95% CI, 27.8-41.2) in pediatric obesity clinics (2).

A long-term, retrospective, cohort study showed that children with NAFLD had a 14 times higher risk of progression to severe liver disease or death when compared to those without (3). NAFLD-related end-stage liver disease is the the leading indication for liver transplantation in adult females (4) and the second most common indication for liver transplantation in all young adults in the United States, expected to become the first by 2030 (5). Since it is often asymptomatic, NAFLD diagnosis might be missed in children (2).

In 2007, the American Academy of Pediatrics (AAP) released guidelines that recommended children aged 9–11 who are obese (body mass index [BMI] ≥ 95th percentile) or overweight (BMI 85th–94th percentile) but presenting also risk factors including central adiposity, insulin resistance, prediabetes or diabetes, dyslipidemia, sleep apnea, and family history of liver disease, should undergo blood tests for aspartate aminotransferase, alanine aminotransferse (ALT), fasting glucose level, and lipid panel to check for complications related to overweight and obesity including the risk for NAFLD development (5).

Guidelines were then published by the North American Society for Pediatric Gastroenterology, Hepatology, and Nutrition (NASPGHAN) in 2017 (6) to further assist physicians who encounter suspected NAFLD in general pediatric offices. This includes persistently (>3 months) elevated ALT, more than twice the upper limit of normal, that should be evaluated for NAFLD or other causes of chronic hepatitis, whereas clinically available routine ultrasound is not recommended as a screening test for NAFLD in children due to inadequate sensitivity and specificity (6).

Poor guideline adherence is a common barrier to screen, identify, and care of patients with chronic conditions, as previously described for asthma and sickle cell disease (7-10). A multicentric study was recently published by Kaiser Permanente and found that ALT screening by AAP guidelines was performed in only 54.0% of children with obesity from 2009 to 2018 (11), indicating that the true incidence of NAFLD is likely not accurate as many patients are likely undiagnosed. However, adherence to the NASPGHAN guidelines has not been so far investigated. Thus, following the NASPGHAN guidelines, we designed and administered a survey to residents enrolled in accredited Pediatric Residency Programs in the United States to assess the consistency of the screening for NAFLD, one of the complications of obesity, and to determine the residents’ extent of training and competence in NAFLD care. By understanding pediatric resident practices, we can understand and address the gaps in medical education regarding NAFLD, the leading indication for liver transplantation in adult women, to hopefully contribute to reduce the incidence rate of this chronic, preventable condition and avoid outcomes such as cirrhosis or transplantation.

## METHODS

### Survey Design, Administration, and Recruitment Strategy

The survey was designed, collected, and managed using REDCap electronic data capture tools (12) hosted at the University of Chicago. According to the NASPGHAN guidelines, “normal” ALT was defined as below 22 mg/dL for girls and 26 mg/dL for boys and “abnormal ALT” was defined as two times these levels, while “Abnormal ultrasound” was defined as “steatosis” or “increased echogenicity” found in liver. After reviewing the initial data, we added one more section of four questions to explore the barriers that residents may face in pursuing the recommended processes for screening and counseling patients with suspected NAFLD. A pilot testing survey was carried out among current postgraduate year PGY-2 and PGY-3 residents at the University of Chicago pediatric residency program before conducting the study nationally. The study was approved and reviewed by the University of Chicago Institutional Review Board.

The survey was emailed five times from May to December 2018 to 206 program directors and residency coordinators at accredited Pediatric Residency US Programs identified in the American Medical Association directory. We requested the survey to be forwarded to upper level pediatrics and medicine/pediatrics (PGY-2, PGY-3, and PGY-4 in combined medicine/pediatrics residents) and offered an option of enrollment in a $25 Visa gift card raffle.

### Data Management and Statistical Analysis

All Likert-scale responses were translated into ordinal numbers: always = 1, often = 2, sometimes = 3, never = 4 or none = 1, minimal = 2, moderate = 3, significant = 4, and they are presented as means ± standard deviations. Categorical variables are presented as total numbers (percentages). To understand if the duration of training impacted the responses, differences between PGY-2 and PGY-3/4 residents for the different survey questions were calculated. Categorical variables were assessed using Chi-square test, while continuous variables, including Likert-scale data, were assessed by t-test (13). A *P* value ≤0.05 was considered statistically significant.

## RESULTS

We estimated that an email with the survey reached 1170 residents based on email receipts (opened, unread, or deleted status) and location data (city, state) reported by participants. Of those, we received 483 surveys from residents of at least 22 states, representing all four US regions (Northeast, Midwest, South and West), resulting in a 41.3% response rate. We evaluated the responses for 399 of 483 participants who fully completed the survey. Eighty-four surveys were removed from the analysis because of incomplete responses. Of the 399 completed surveys, 269 participants filled out the additional education and barrier questions. Among the respondents, 178 (44.6%) were PGY-2 while 221 (55.4%) were PGY-3/4 residents (Table 1). We did not compare responses between pediatric versus medicine-pediatrics residents given much fewer number of responses from the latter group. Similarly, we did not compare responses based on residency setting since the number of respondents from university-based programs significantly exceeded those from community-based ones (68.9% vs. 31.1%, Table 1). Patient population in clinics often included overweight or obese children: 49.9% (199/399) residents reported encountering 26–50% overweight or obese patients, whereas 38.4% encountered more than 50% of them (Table 1).

**TABLE 1.** Participant characteristics

	PGY-2 n (%)	PGY-3/4 n (%)	Total n (%)	*P* *
No. of participants	178 (44.6)	221 (55.4)	399 (100.0)	
**Training program**				10.25
Pediatrics	173 (97.2)	196 (88.7)	369 (92.5)	
Medicine-pediatrics	5 (2.8)	25 (11.3)	30 (7.5)	
**Percent of clinic patients BMI >85%ile**				0.068
<25%	26 (14.6)	21 (9.5)	47 (11.8)	
26–50%	83 (46.6)	116 (52.5)	199 (49.9)	
51–75%	66 (37.1)	72 (32.6)	138 (34.6)	
>75%	3 (1.7)	12 (5.4)	15 (3.8)	
**Future career path**				0.029
Primary care	51 (28.7)	79 (35.7)	130 (32.6)	
Hospitalist	18 (10.1)	31 (14.0)	49 (12.3)	
Subspecialty	89 (50.0)	101 (45.7)	190 (47.6)	
Undecided	20 (11.2)	10 (4.5)	30 (7.5)	
**Residency setting**				0.552
University-based	119 (66.9)	156 (70.6)	275 (68.9)	
Community-based, university affiliate	41 (23.0)	49 (22.2)	90 (22.6)	
Community-based, no university-affiliate	18 (10.1)	16 (7.2)	34 (8.5)	

*P value based on chi-square test, comparing distributions between PGY-2 and PGY-3/4.

BMI = Body mass index.

NASPGHAN guidelines suggest to screen all obese children aged 9–11 and overweight children with risk factors (central adiposity, insulin resistance, pre or diabetes, dyslipidemia, sleep apnea), or younger patients with risk factors, including severe obesity, family history of NAFLD/NASH, or hypopituitarism. First-degree relatives of children with NAFLD should also be screened.

**FIGURE 1.**
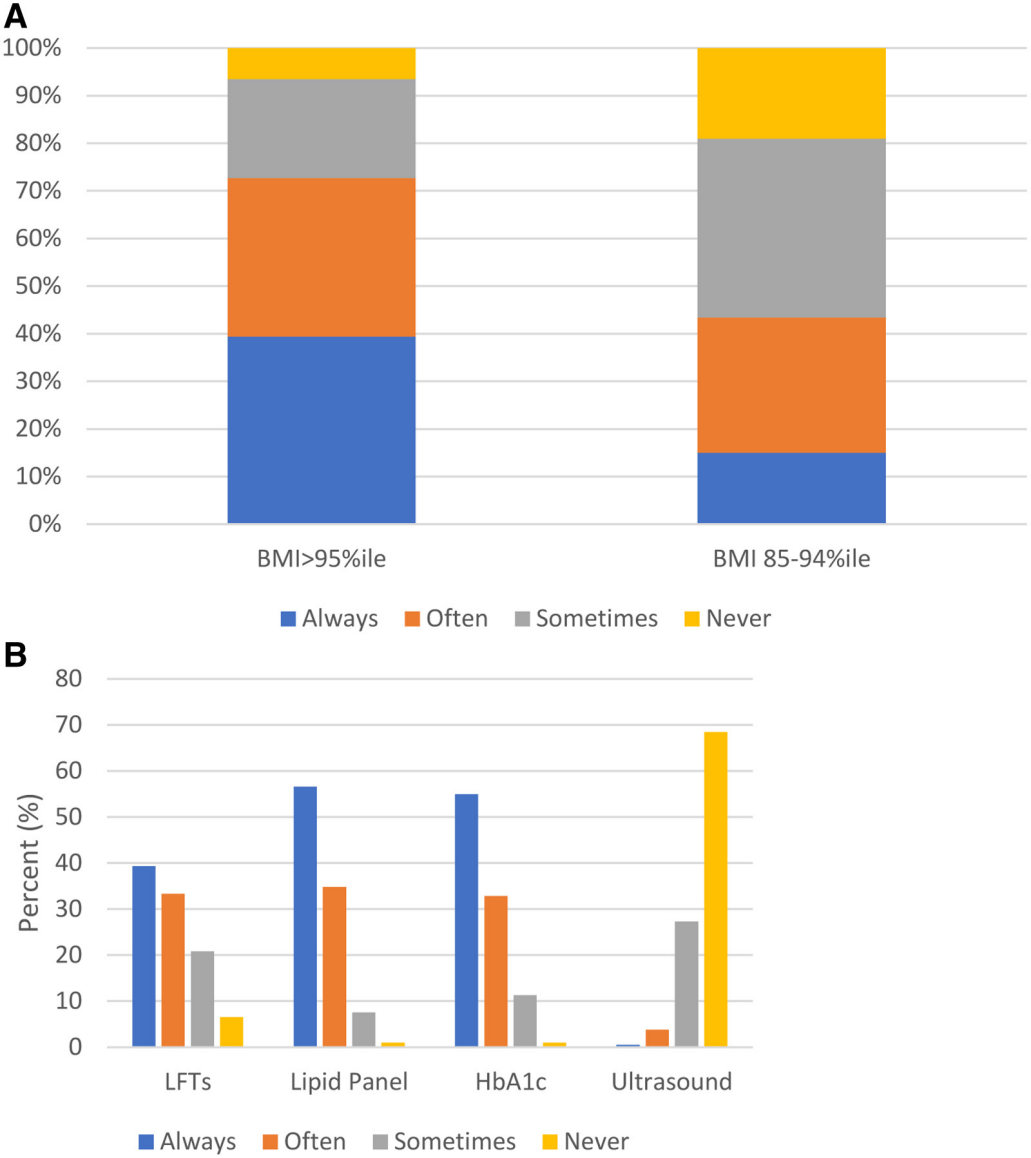
Frequency of obtaining liver chemistry panel in screening for NAFLD. A) Comparing liver chemistry checks when screening obese patients (BMI > 95th percentile) versus overweight (BMI 85–94th percentile). B) Comparing frequency of checking LFTs to other tests, *P* < 0.01 using paired t-test. BMI = body mass index; LFTs = liver function tests; NAFLD = nonalcoholic fatty liver disease.

The first section of the survey assessed the screening process of overweight and obese pediatric children by the residents for NAFLD. We first asked how often residents check growth parameters and inquire about personal and family history that might unveil comorbidities or risk factors for obesity and related complications (see Supplemental Digital Content Table 1, http://links.lww.com/PG9/A88). When inquired about patients with BMI >95th percentile, 39% consistently ordered liver function tests, while 6.5% never ordered them. Comparably, in overweight patients (BMI 85th–94th percentile), screening was appropriately performed less often with the majority of residents (38%) reporting sometimes (*P* < 0.01, Fig. 1A).

We investigated the factors that led residents to initiate evaluation for NAFLD. Although the NASPGHAN guidelines describe that the prevalence of NAFLD increases by increasing BMI, age, male gender, Hispanic ethnic background, the residents indicated age, BMI, and family history of liver disease as decision-making factors for NAFLD evaluation, not recognizing race/ethnicity and gender (see Supplemental Digital Content Table 2, http://links.lww.com/PG9/A88).

According to the NASPGHAN guidelines the best screening test for NAFLD in children is ALT, whereas routine liver ultrasound is not recommended due to inadequate sensitivity and specificity (7), despite its importance in the diagnostic workup of persistently elevated liver enzymes to rule out other causes. Residents ordered an ALT more often than ultrasound for screening purposes (Fig. 1B). In the context of screening for obesity-related comorbidities, other metabolic tests, such as lipid panel and HbA1c, were more likely to be ordered (Fig. 1B) compared with ALT. PGY-2 residents were overall less likely to order screening tests than PGY-3,4 residents but there was no statistically significant difference (data not shown). Despite ordering screening ALT, respondents were unlikely to discuss NAFLD as a comorbidity of obesity (see Supplemental Digital Content Figure 1, http://links.lww.com/PG9/A88), independent of year of training.

We then assessed residents’ decision-making process upon receiving the results from the initial evaluation. NASPGHAN recommends lifestyle modifications, including diet and increased physical activity, as the first-line treatment for all children with NAFLD, repeating ALT every 2 to 3 years if initially normal and risk factors remain unchanged, whereas sooner if clinical risk factors increase in number or severity (6). In line with the recommendations, over half of respondents (52.6%) indicated “counseling about healthy lifestyle modification and repeat labs in 2 or more years” as the next management step for obese patients with normal ALT (Table 2). The level of training did not impact this choice (*P* = 0.514).

**TABLE 2.** Management of NAFLD

	PGY-2	PGY-3/4	Total	*P*
If your patient has a normal ALT, what is your next step?	**n (%**)	**n (%**)	**n (%**)	0.078
Nothing	5 (2.8)	6 (2.7)	11 (2.8)	
Counsel and repeat in 2+ y	99 (55.6)	111 (50.2)	210 (52.6)	
Counsel and repeat in 6 mo to 1 y	67 (37.7)	78 (35.3)	145 (36.3)	
Refer to a weight management clinic	7 (3.9)	24 (10.9)	31 (7.8)	
Order a liver ultrasound	0 (0)	2 (0.9)	2 (0.5)	
Are patients with normal ALT still at risk for NAFLD?				0.514
Yes	168 (94.4)	205 (92.8)	373 (93.5)	
If your patient has an elevated ALT, what is your next step?				0.017
Counsel on lifestyle modification	39 (21.9)	43 (19.5)	82 (20.6)	
Follow-up in 1–3 mo	11 (6.2)	15 (6.8)	26 (6.5)	
Repeat labs immediately	24 (13.5)	14 (6.3)	38 (9.5)	
Obtain liver ultrasound	56 (31.5)	57 (25.8)	113 (28.3)	
Refer to pediatric gastroenterology	38 (21.3)	79 (35.7)	117 (29.3)	
Refer to a weight management clinic	10 (5.6)	13 (5.9)	23 (5.8)	
When do you refer to pediatric gastroenterology?	**n (%**)	**n (%**)	**n (%**)	0.158
Elevated ALT	46 (25.8)	79 (35.7)	125 (31.3)	
Abnormal ultrasound	56 (31.5)	60 (27.2)	116 (29.1)	
Elevated ALT and abnormal ultrasound	71 (39.9)	79 (35.7)	150 (37.6)	
Normal workup but increasing weight	5 (2.8)	3 (1.4)	8 (2.0)	

n (%): number (percentage) of residents who responded yes. *P* value based on chi-square test comparing distributions between PGY-2 and PGY-3/4.

ALT = alanine aminotransferse; NAFLD = nonalcoholic fatty liver disease.

On another hand, the approach to children with elevated ALT values considerably deviated from the guidelines. NASPGHAN recommends repeat ALT in 3 months with further evaluation if persistent elevation and underemphasized the role of ultrasonography for NAFLD screening. Only 6.5% of residents chose to re-evaluate in 3 months although 9.5% did chose to re-test immediately. The majority was split between referring to Pediatric Gastroenterology/Hepatology clinic (29.3%), obtaining liver ultrasound (28.3%), and healthy lifestyle counseling (20.6%). Level of training impacted this choice, in fact PGY-3/4 residents were more likely than PGY-2 residents to refer to Pediatric Gastroenterology, whereas less likely to repeat lab testing (*P* = 0.017, Table 2).

Even though most residents accurately recognized the risk of NAFLD despite normal ALT levels (93.5%, Table 2), it was infrequently discussed, while significantly more likely discussed with patients with elevated ALT (see Supplemental Digital Content Figure 1, http://links.lww.com/PG9/A88). In those that reported counseling on NAFLD with patients, cirrhosis was rarely discussed with 23.6% reporting to “Never” discuss it and majority (33.7%) discussing it only “Sometimes” (data not shown).

Residents were likely to counsel patients on changes in diet (45.3% reporting “Always”) and exercise (67.1% reporting “Always,” data not shown), which are the first-line recommendations by the NASPGHAN (6).

According to the NASPGHAN, ALT levels of 22 mg/dL for girls and 26 mg/dL for boys were considered normal, whereas abnormal ALT was defined as two times these levels, whereas abnormal liver ultrasound refers to “steatosis” or “increased liver echogenicity” (6). Referral to the pediatric gastroenterologist or hepatologist is suggested for symptomatic patients with abnormal ALT or those with ALT > 80 U/L or persistent ALT elevation despite lifestyle modification. Only 31.3% of residents referred to a Pediatric Gastroenterologist for solely abnormal ALT, whereas 37.6% choose to refer if both ALT and liver ultrasound were abnormal, 29.1% for solely abnormal ultrasound, whereas only 2% for continued weight gain independently of lab tests results (Table 2). PGY-3/4 more often than PGY-2 residents referred for an elevated ALT without imaging (*P* = 0.158, Table 2).

In the last section of the survey, we inquired about education and training on NAFLD. As for NAFLD-related education, the majority of residents (68.4%) reported not receiving any lecture on NAFLD during their training, and 85.5% not feeling comfortable with the level of education they had about NAFLD, independently from the year of training (Table 3). Among those who received training on the topic (31.6%), this was most often delivered in the form of a formal lecture (75.4%) rather than in clinic discussion (15.1%) or during rounds (9.5%). When asked about the preferred format, more than half of the responding residents marked formal lecture as the preferred method (56.1%) to implement their education on the topic. When exploring the barriers residents face in screening and counseling patients with suspected NAFLD, understanding of disease process (knowledge) was the biggest barrier (25.7%), followed by training experience (22.7%) with no statistically significant difference (*P* = 0.56). Comparatively, comfort level (personal level of psychological ease in approaching and discussing obesity/comorbidities including NAFLD) was ranked significantly lower although still impacted residents’ management (Table 3).

**TABLE 3.** Education on NAFLD

	PGY-2	PGY- 3/4	Total	*P*
Have you been given a talk on NAFLD?^*^				
	**mean** ± **SD**	**mean** ± **SD**	**mean** ± **SD**	0.163
Yes	51 (28.7)	75 (33.9)	126 (31.6)	
No	127 (71.3)	146 (66.1)	273 (68.4)	
If yes, what setting were you given a talk?^*^				
	**n (%**)	**n (%**)	**n (%**)	0.033
Formal lecture	34 (66.7)	61 (81.3)	95 (75.4)	
During rounds	9 (17.6)	3 (4)	12 (9.5)	
At clinic	8 (15.7)	11 (14.7)	19 (15.1)	
Do you feel comfortable with the level of education you have received?^*^				
	**n (%**)	**n (%**)	**n (%**)	0.323
Yes	13 (11.9)	26 (16.2)	39 (14.5)	
No	96 (88.1)	134 (83.8)	230 (85.5)	
What is your educational format preference to learn about NAFLD?^*^				
	**n (%**)	**n (%**)	**n (%**)	0.918
Formal Lecture	64 (58.7)	87 (54.4)	151 (56.1)	
During rounds	12 (11)	19 (11.9)	31 (11.5)	
During continuity clinic	23 (21.1)	38 (23.7)	61 (22.7)	
Workshop	10 (9.2)	16 (10)	26 (9.7)	
Ranking of barriers residents face in screening and counseling patients for NAFLD^†^				
	**mean** ± **SD**	**mean** ± **SD**	**mean** ± **SD**	
Training	2.89 ± 0.76	2.94 ± 0.80	2.92 ± 0.78	0.577
Knowledge	2.93 ± 0.82	2.96 ± 0.77	2.94 ± 0.79	0.764
Competency	2.73 ± 0.75	2.69 ± 0.82	2.71 ± 0.80	0.685
Comfort	2.46 ± 0.82	2.36 ± 0.88	2.40 ± 0.86	0.336

^*^n (%) who responded yes. *P* value based on chi-square test.

^†^Mean ± SD. Likert-scale responses: always = 1, often = 2, sometimes = 3, never = 4. *P* values based on 2-sided t-test.

NAFLD = nonalcoholic fatty liver disease.

## DISCUSSION

This is the first study to assess the current state of pediatric NAFLD screening and management among residents across the United States in different types of pediatric residency program (academic vs. community based). Although pediatric residents are frequently exposed to pediatric obesity in their continuity clinics with over 88% reporting that more than 25% of their patients in continuity clinic are overweight or obese, our results suggest that they lack training in the evaluation and management of NAFLD, the second leading, yet preventable, cause of liver transplantation in adults.

Our survey reveals that a good rate of residents screened overweight and obese pediatric patients by routinely checking liver enzymes, although less commonly than HbA1c or lipid panel, requested to identify other obesity-related comorbidities. Despite 93.5% of residents acknowledged that NAFLD was a potential complication for obese patients, they were very unlikely to mention it as a comorbidity. Notably, discussing the NAFLD spectrum was lacking even with patients with elevated ALT, with 85.5% of residents reporting to feel uncomfortable discussing it, indicating that some degree of discomfort was reported regardless of signs of liver injury. Likely a combination of unpreparedness, lack of knowledge and inadequate training contributed to generate such discomfort.

Race/ethnic background and gender were not correctly identified as major risk factors for NAFLD despite a well-documented increased risk among Hispanic and males (2), and notably, family history of liver disease was rarely investigated. These data, along with 68.4% residents reporting not having received any education on NAFLD, indicate that residents are unprepared on its management. This prompted us to further explore this topic with additional questions.

These were based on a proposed conceptual model for potential factors that impact the performance of pediatric residents (14). It is mainly shaped by the interaction of 4 educational domains: training experience, acquired personal fund of knowledge, achieving competency in performing the evaluation and counseling, and the comfort level and psychological ease that the residents build up through appropriate training. These domains are adapted for NAFLD from Kolarik et al in their assessment of pediatric residents’ education and training in the area of palliative care.

Both PGY-2 and PGY-3/4 residents reported inadequate training in all the different educational domains. This was perceived to impact their basic fund of knowledge, competency in performing the evaluation and counseling of patients with suspected NAFLD, and their comfort and psychological ease in approaching and discussing obesity as a medical disease along with its comorbidities including NAFLD. Knowledge was ranked the highest barrier to screening and counseling patients with NAFLD, followed by inadequate training and low competency. Comfort with screening and counseling seem to be the least impactful barrier, suggesting that they felt prioritizing knowledge, training, and competency.

The responsibility of preventing obesity and its comorbidities is a shared task among all elements of the society. Primary care providers often bear the responsibility given their first-line relationship with families and patients. Their potentially influential position to attract broader community support should be valued and their education implemented, especially in a scenario characterized by an overwhelming increase of guidelines published yearly. In addition, we must advocate that pediatricians of all specialties share the role of addressing this growing problem and adhere to newly published guidelines. In this context, education on obesity and its comorbidities, including NAFLD, and associated guidelines should be adequately covered in residency programs and fully integrated in the education core curriculum of every pediatric and family practice residency in the country. The American Medical Association developed learning objectives to teach chronic disease prevention and management to medical students (17) that might be incorporated with the modules developed by the AAP (18), to educate residents and primary care providers on how to discuss obesity and its comorbidities. We believe that such modules and learning objectives should be implemented to include a focus on the NAFLD spectrum and other obesity-related comorbidities in pediatric clerkships and in the outpatient curriculum for residency programs around the country.

Importantly, over 50% of residents reported preferring formal lectures over other teaching approaches, to enhance their educational opportunities on NAFLD. Nonetheless, we believe these formal approaches should be integrated with more effective *in person* training and targeted supervision during daily practice, possibly by clinic preceptors, to improve management and counseling with patients and families for all obesity-related comorbidities, including NAFLD, while avoiding the risk of residents’ burn out as a result of increasing training hours.

One limitation of this study is that there is potential for sampling and nonresponse bias since we are unable to truly determine which and how many residents received the survey given that we relied on program directors and program coordinators to forward our survey email to their pediatric and medicine/pediatrics residents and are unable to survey those who did not initially respond. It is known that surveying physicians is a major challenge, especially for web-based survey, with a decline in the past half century of the response rate to be around 40–50% (13). Busy schedules, frequent survey requests and fear of responses being used against them have been cited as barriers (15,16). Minor limitations were also the lack of definition of “abnormal” ultrasound in survey question no. 16 and the lack of clarification that liver function tests were requested to screen for NAFLD in survey question no. 5. Despite these limitations, this study represents the first survey in the United States to test the adequacy of residents’ training practices in adherence to pediatric NAFLD guidelines.

In conclusion, the results of this survey indicate that residents perceived themselves to be inadequately trained on pediatric NAFLD, this resulted in poor counseling and inappropriate management of children at risk for NAFLD. Implementing dedicated time to discuss NAFLD in the context of the latest guidelines during residency training has become a necessity to achieve a higher degree of competence and comfort in the management of an increasingly common and morbid disease.

Specific educational interventions should be pursued based on residents’ perceived needs with the goal of implementing enhanced, updated, and multifaceted educational policies to improve early screening, evaluation, and management of overweight and obese children at risk for NAFLD in accordance with published guidelines.

## ACKNOWLEDGMENTS

Dr. Azzam conceived and designed the study. Dr. Azzam mentored Dr Patel to create the materials. Dr. Azzam executed the study. Nour Asfour performed the statistical analysis. Dr Discepolo, Dr. Azzam, and Dr Patel contributed to the interpretation of the data, writing, and editing the article. All authors approved the final article as submitted and agree to be accountable for all aspects of the work.

## Supplementary Material


