# Supplementary material for: Preparedness of Residents to Manage Pediatric Nonalcoholic Fatty Liver Disease: A National Survey
Source: JPGN Rep. 2022 Jun 21;3(3):e219. doi: 10.1097/PG9.0000000000000219 (PMC9881435; doi:10.1097/PG9.0000000000000219)
Supplement: Supplementary file 1 [file pg9-3-e219-s001.pdf]

Supplemental Table 1. Initial Screening and Discussion of NAFLD.

	**PGY-2**	**PGY-3/4**	**Total**	***p*-value**
	**mean +/- SD**	**mean +/- SD**	**mean +/- SD**	
**If patient's BMI > 85%ile, how often do you assess:**				
Growth Parameters	1.08 +/- 0.29	1.10 +/- 0.32	1.09 +/- 0.31	0.599
Diet History	1.29 +/- 0.51	1.25 +/- 0.48	1.27 +/- 0.49	0.504
Exercise	1.37 +/- 0.57	1.38 +/- 0.58	1.37 +/- 0.57	0.797
Screen Time	1.82 +/- 0.82	1.86 +/- 0.77	1.84 +/- 0.79	0.582
Snoring	2.33 +/- 0.78	2.32 +/- 0.85	2.32 +/- 0.82	0.912
Hypertension	1.70 +/- 0.84	1.57 +/- 0.70	1.63 +/- 0.77	0.088
Family History of Obesity	2.48 +/- 0.96	2.45 +/- 0.88	2.47 +/- 0.92	0.740
Family History of Dyslipidemia	2.46 +/- 0.96	2.52 +/- 0.93	2.49 +/- 0.95	0.464
Family History of Type 2 Diabetes	2.04 +/- 0.89	2.19 +/- 0.86	2.13 +/- 0.87	0.091
Family History of Hypertension	2.11 +/- 0.90	2.36 +/- 0.90	2.25 +/- 0.90	0.006
Family History of Liver Disease	3.10 +/- 0.81	3.04 +/- 0.89	3.06 +/- 0.86	0.494
**Do you discuss NAFLD as a co-morbidity?**				
	2.63 +/- 1.05	2.70 +/- 1.04	2.67 +/- 1.04	0.520
**Does this factor affect your decision in initiating evaluation of NAFLD?**				
	**n(%)**	**n(%)**	**n(%)**	***p*-value**
BMI	166 (93.3)	204 (92.3)	370 (92.7)	0.72
Age	114 (64)	135 (61.1)	249 (62.4)	0.54
Gender	25 (14)	20 (9)	45 (11.3)	0.12
Race/Ethnicity	49 (27.7)	57 (25.8)	106 (26.6)	0.67
Family History of Obesity	89 (50)	112 (50.7)	201 (50.4)	0.89
Family History of Liver Disease	126 (70.8)	155 (70.1)	281 (70.4)	0.89

Mean +/- Standard Deviation. Likert scale responses: always=1, often=2, sometimes=3, never=4. *p*-values based on 2-sided t-test.

Supplemental Table 2. Risk Factors of NAFLD.

Does this factor affect your decision in initiating evaluation of NAFLD?				
	**n(%)**	**n(%)**	**n(%)**	***p*-value**
BMI	166 (93.3)	204 (92.3)	370 (92.7)	0.72
Age	114 (64)	135 (61.1)	249 (62.4)	0.54
Gender	25 (14)	20 (9)	45 (11.3)	0.12
Race/Ethnicity	49 (27.7)	57 (25.8)	106 (26.6)	0.67
Family History of Obesity	89 (50)	112 (50.7)	201 (50.4)	0.89
Family History of Liver Disease	126 (70.8)	155 (70.1)	281 (70.4)	0.89

n (%) who responded yes. *p*-value based on chi-square test

Supplemental Figure 1. Discussion of NAFLD.

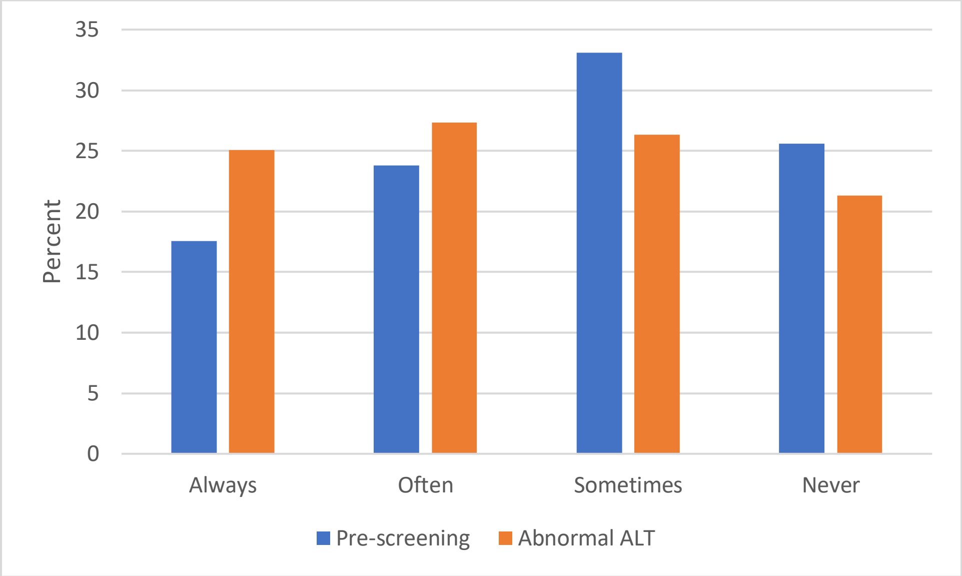

p<0.01 using paired t-test

Are Pediatric Residents Prepared to Evaluate and Initiate Management of Children with Suspected Non-Alcoholic Fatty Liver Disease: A National Survey Study

Instructions:
Please choose ONE answer to every question or subcategory question.

Part 1 assesses the screening process of overweight and obese pediatric children by the residents for NAFLD:

1. Do you review growth parameters measurements and percentiles (weight, height and BMI) when you see a pediatric patient in clinic:
   a. Yes, every time ……………………. 1
   b. Yes, but not every time …………… 2
   c. Never …………………… 3

2. In patients who are overweight (BMI >85th %ile and < 95th %ile) or obese (BMI ≥95th%ile) do you assess:

	Yes, every time	Yes, but not every time	Never
a. Dietary history	1	2	3
b. Exercise	1	2	3
c. Screen time	1	2	3
d. Hypertension	1	2	3
e. Snoring	1	2	3
f. Family Hx of obesity	1	2	3
g. Family Hx of dyslipidemia	1	2	3
h. Family hx of T2DM	1	2	3
i. Family Hx of HTN	1	2	3
j. Family Hx of liver disease	1	2	3

3. Do you discuss the co-morbidity of Non-Alcoholic Fatty Liver Disease with your overweight or obese pediatric patients and their families?

a. Yes, every time …………………………….. 1
b. Yes, but not every time …………………….. 2
c. Never ……………..3

Part 2 assesses the evaluation process that residents follow to evaluate for NAFLD:

4. What affects your decision in initiating evaluation for NAFLD?

	Yes	No
a. BMI	1	2
b. Age	1	2
c. Gender	1	2
d. Race/ethnic background	1	2
e. Obesity in family	1	2
f. Family Hx of liver disease	1	2

5. In patients who are obese (BMI > 95tht^{h} %ile) do you order:

	Yes, every time	Yes, but not every time	Never
a. LFTs	1	2	3
b. Lipid panel	1	2	3
c. HbA1C	1	2	3
d. TSH	1	2	3
e. Liver ultrasound	1	2	3

6. In patients who are overweight (BMI >85th %ile and < 95th %ile) do you check LFTs:
   a. Yes, every time …………………………………………………… 1
   b. Yes, but not every time ……………………………………………. 2
   c. Never …… 3

Part 3 assesses the decision making after some evaluation tests have been performed

7. For obese patients with normal ALT, what is your next management step:
   a. Nothing
   b. Counsel about healthy lifestyle modification and never repeat
   c. " Counsel…" and repeat it in 6 months – 1 year

d. " Counsel…" and repeat it in > 1 year
e. Order a liver ultrasound
f. Refer to a weight management program
g. Refer to the Pediatric Gastroenterology clinic

8. For patients who are overweight/obese with an initial normal ALT, do you believe this patient is no longer at risk for NAFLD?
   a. Yes …………………………. 1
   b. No …………………………. 2

9. For obese/overweight patients with abnormal ALT, do you discuss the possibility of NAFLD?
   a. Yes, every time …………………………….. 1
   b. Yes, but not every time …………………….. 2
   c. Never (skip to Q 15) ……………….. 3

10. When you discuss NAFLD, do you inform the patient/family that it is a spectrum of disease with cirrhosis/scarring of the liver at the severe end of the spectrum?
    a. Yes, every time ………………………….. 1
    b. Yes, but not every time …………………… 2
    c. Never …………………………… 3

11. For overweight or obese patients with an elevated ALT, what is your next management step:
    a. Counsel about healthy lifestyle modification
    b. Close follow up
    c. Repeat the lab
    d. Request a liver ultrasound
    e. Refer to Pediatric Gastroenterology
    f. Refer to a weight management program

Part 4 assesses the anticipatory guidance residents provide to overweight/obese patients and their families:

12. Do your overweight/obese patients get dietary counseling at your clinic?
    a. Yes, every time ………………………. 1
    b. Yes, but not every time ………………….. 2
    c. Never (skip to Q 18) ……………. 3

13. Who provides this counseling?
    a. Me (resident)
    b. Supervising attending
    c. Dietician in clinic

14. What kind of diet do you or the clinic dietitian counsel patients/families on?
    a. Low calorie ……………………. 1
    b. Low carbohydrate ……………... 2
    c. Low fat ………………………... 3
    d. Only no fructose ………………… 4

15. Do you counsel overweight/obese patients/families on exercise?
    a. Yes, every time ……………………………… 1
    b. Yes, but not every time ………………………. 2
    c. Never ………………………………. 3

Part 5 assesses for the Pediatric Liver Clinic referral decision making:

16. When do you refer overweight/obese patients to Pediatric Gastroenterology?
    a. If ALT is not normal ……………………………………1
    b. If liver ultrasound is not normal ………………………... 2
    c. If both ALT and liver ultrasound are abnormal ………… 3
    d. If labs are normal but weight continues to rise ………… 4

Part 6 assesses the education residents receive on NAFLD:

1. Have you been given a talk on NAFLD?
    a. Yes
    b. No (skip question 2)

2. What setting?
    a. Formal lecture
    b. During rounds
    c. At continuity clinic
3. Do you feel comfortable with the level of education you have received on NAFLD?
    a. Yes
    b. No

4. Rank barriers you face in screening and counseling NAFLD patients, 1= not a barrier, 2= minimal, 3= moderate, 4= significant
    a. Training experience (inadequate teaching on NAFLD) 1 2 3 4
    b. Knowledge (personal fund of knowledge on NAFLD) 1 2 3 4
    c. Competency (ability to perform evaluation and counseling of suspected NAFLD)
    1 2 3 4
    d. Comfort (personal level of psychological ease in approaching and discussing obesity/ comorbidities including NAFLD) 1 2 3 4

5. What is your preference on format for educating residents on NAFLD:
    a. Formal lecture
    b. During rounds
    c. During continuity clinic
    d. Workshop

Part 7: Demographics of the respondents:

1. Your training:
    a. Pediatrics
    b. Medicine/ Pediatrics

2. Year of Pediatric residency training you are in, PGY- :
    a. 2
    b. 3 or 4

3. What percentage of patients you see in your continuity clinic do you think are overweight or obese?
    a. ≤25%
    b. 26- 50%
    c. 51-75%
    d. > 75%

4. What are your future plans?
    a. Primary Care
    b. Hospitalist
    c. Sub-specialty
    d. Undecided

End of Survey

Thank you for taking the survey.

The prevalence of NAFLD parallels the prevalence of obesity in children and is currently 29-38% in obese children.

NAFLD can lead to inflammation, fibrosis and then cirrhosis of the liver which can lead to need for liver transplant and death. Lifestyle change (healthier diet with increased physical activity) has proven to be effective at slowing down the progression of disease.

Please review the NASPGHAN guidelines today!

https://www.naspghan.org/files/NASPGHAN_NAFLDClinical_Practice_Guideline.pdf

Have a nice day!

Email confirmation
We appreciate your participation in this study!

Please click on this link and provide your email address if you would like to participate in a raffle to win a $25 Visa gift card: https://goo.gl/forms/qE93JACCYNlnjhZO2

The purpose of a separate link is to disconnect your email address from your survey responses.

Namrata Patel, MD, MPH
Pediatrics Resident
University of Chicago Comer Children's Hospital

Ruba Azzam, MD
Pediatric Hepatologist
University of Chicago Comer Children's Hospital
